# Alkane inducible proteins in *Geobacillus thermoleovorans *B23

**DOI:** 10.1186/1471-2180-9-60

**Published:** 2009-03-25

**Authors:** Tomohisa Kato, Asuka Miyanaga, Shigenori Kanaya, Masaaki Morikawa

**Affiliations:** 1 Department of Material and Life Science, Graduate School of Engineering, Osaka University, Osaka 565-0871, Japan; 2 Current Address : Division of Biosphere Science, Graduate School of Environmental Science, Hokkaido University, Sapporo 060-0810, Japan

## Abstract

**Background:**

Initial step of β-oxidation is catalyzed by acyl-CoA dehydrogenase in prokaryotes and mitochondria, while acyl-CoA oxidase primarily functions in the peroxisomes of eukaryotes. Oxidase reaction accompanies emission of toxic by-product reactive oxygen molecules including superoxide anion, and superoxide dismutase and catalase activities are essential to detoxify them in the peroxisomes. Although there is an argument about whether primitive life was born and evolved under high temperature conditions, thermophilic archaea apparently share living systems with both bacteria and eukaryotes. We hypothesized that alkane degradation pathways in thermophilic microorganisms could be premature and useful to understand their evolution.

**Results:**

An extremely thermophilic and alkane degrading *Geobacillus thermoleovorans *B23 was previously isolated from a deep subsurface oil reservoir in Japan. In the present study, we identified novel membrane proteins (P16, P21) and superoxide dismutase (P24) whose production levels were significantly increased upon alkane degradation. Unlike other bacteria acyl-CoA oxidase and catalase activities were also increased in strain B23 by addition of alkane.

**Conclusion:**

We first suggested that peroxisomal β-oxidation system exists in bacteria. This eukaryotic-type alkane degradation pathway in thermophilic bacterial cells might be a vestige of primitive living cell systems that had evolved into eukaryotes.

## Background

Thermophilic bacteria offer crucial advantages over mesophilic or psychrophilic bacteria, especially when they are applied to ex-situ bioremediation processes. Limited biodegradation of hydrophobic substrates caused by low water solubility at moderate temperature conditions can be overcome if the reaction temperature could be increased enough. We previously isolated an extremely thermophilic alkane-degrading bacterium, *Goebacillus thermoleovorans *(previously *Bacillus thermoleovorans*) B23, from a deep-subsurface oil reservoir in Japan [1,2]. Strain B23 effectively degraded alkanes at 70°C with the carbon chain longer than twelve, dodecane. Since tetradecanoate and hexadecanoate or pentadecanoate and heptadecanoate were accumulated as degradation intermediates of hexadecane or heptadecane, respectively, it was indicated that the strain B23 degraded alkanes by a terminal oxidation pathway, followed by β-oxidation pathway. Recently, another long-chain alkane degrading *Geobacillus thermodenitrificans *NG80-2 was also isolated from a deep-subsurface oil reservoir [3] and its complete genome sequence was determined [4].

Besides their biotechnological importance, thermophilic microorganisms maintain interesting features useful for studying evolution of life. Microorganisms living under extremely high temperature condition, such as hyperthermophilic archaea and hyperthermophilic bacteria, share the cellular mechanisms with not only bacteria but also eukaryotes [5,6]. This is consistent with an evolutionary hypothesis based on a phylogenetic analysis of 16S and 18S rRNA genes, that hyperthermophiles are very primitive and are close relatives of the common ancestor of living organisms [7]. Extremely thermophilic bacteria, that grow under deep subterranean environment, would also add knowledge to the evolution of life because the condition at subsurface is regarded to be more stable than the surface of the earth. Although alkane degradation is not a central metabolic pathway of the cells, it would be informative to compare the pathway of thermophilic bacteria with that of mesophilic bacteria and eukaryotes. Since most studies on the alkane degradation pathway have been performed on mesophilic microorganisms, such as *Pseudomonas oleovorans *[8], *Acinetobacter *sp. [9], *Candida tropicalis *[10], and *Yarrowia lipolitica *[11], we decided to study on the alkane metabolisms of extremely thermophilic bacteria. Recently, a unique alkane monooxygenase that belongs to luciferase family was reported for *G. thermodenitrificans *[12].

Here, we report that two novel membrane proteins, superoxide dismutase, catalase, and acyl-CoA oxidase activities were dramatically increased in the cells of *G. thermoleovorans *B23 when they were grown on alkanes. Induction of above enzymatic activities upon alkane degradation has never been reported for bacteria but reported for yeast, such as *C. tropicalis *[13,14]. This result suggests that alkane degradation pathway is at least partly shared by eukaryotes and deep-subsurface thermophilic bacteria.

## Results and Discussion

### Microscopic observations

**Figure 1:**
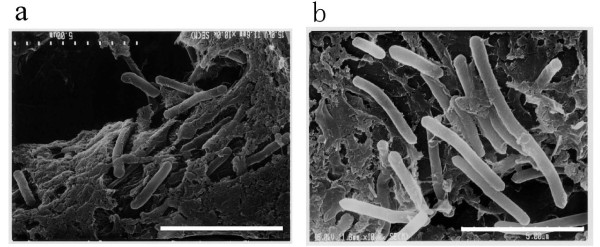
**Scanning electron micrographs of the strain B23 cells before (a) and after (b) cultivation on LBM supplemented with 0.1% (v/v) alkanes**. Cells were grown without shaking at 70°C for 14 days. The bars indicate the size of 5 μm. Background of the cells is cellulose fibers of filter paper on which cells are adsorbed and fixed.

The shape of *G. thermoleovorans *B23 cells before and after cultivation in the presence of alkanes was compared with each other by a scanning electron microscope (Fig. 1a, b). It was found that the cells became longer and thicker after 14-day growth on alkanes. No such swell was observed for the cells grown in the absence of alkanes (picture not shown). This dynamic change of cell shape prompted us to analyze the cellular proteins produced in relation to alkane degradation.

### Induction of protein productions by alkanes

**Figure 2:**
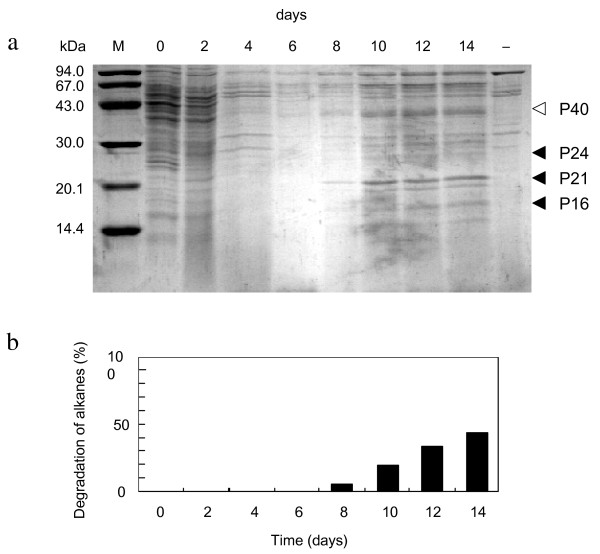
**a, Induction of P24, P21 and P16 productions in *G. thermoleovorans *B23**. Cells were cultivated in LBM supplemented with 0.1% alkane mixtures (V/V) for 14 days at 70°C. Total cell fractions were loaded on an SDS-12% polyacrylamide gel. Protein bands were stained with Coomassie Brilliant Blue R-250. Lanes are molecular size marker, M; cultures after 0 day, 0; 6 days, 6; 8 days, 8; 10 days, 10; 12 days, 12; 14 days, 14; and 14 days cultivation in the absence of alkanes, -. b, Relative degradation of alkanes by strain B23. Fractions degraded were estimated by the reduction of peak areas in GC/FID.

**Figure 3:**
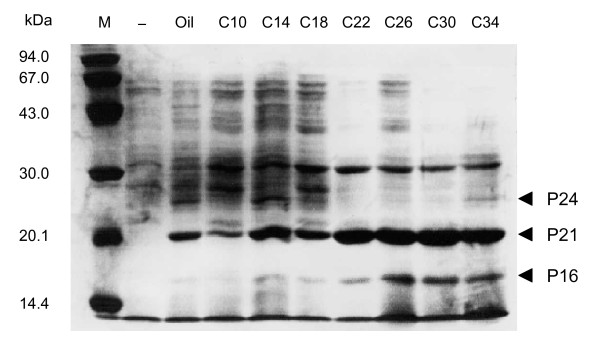
**Effects of long-chain alkanes on the induction levels of P24, P21 and P16**. Proteins were separated on an SDS-12% polyacrylamide gel and stained with Coomassie Brilliant Blue R-250. Lanes are molecular size marker (M), total cellular proteins in the absence of alkanes (-); total cellular proteins in the presence of decane (C10), tetradecane (C14), octadecane (C18), docosane (C22), hexacosane (C26), triacontane (C30), tetracontane (C34).

Comparative analysis of proteins by SDS-PAGE showed that production levels of at least three kinds of proteins were increased after 10-day cultivation with alkanes (Fig. 2a). These were 24 kDa, 21 kDa and 16 kDa proteins, which were designated as P24, P21 and P16, respectively. Although a protein band at 40 kDa (P40) also seems to increase in Fig. 2a, reappearance of this phenomenon was not high (see Fig. 3) and therefore no further work was performed on this protein. When the cells were simultaneously exposed to alkanes in rich nutrient L-broth, where catabolite repression would have probably prevented alkane degradation gene from being expressed, induction of these proteins were not observed. It is of interest that increase in the production level of these three proteins became significant at the time when alkane degradation started (Fig. 2b). When we tested other hydrophobic substrates, no such induction was observed for palmitic acid, tributyrin, trimyristin, or dicyclopropylketone (DCPK) which is an inducer of alkane degradation gene expression in *P. oleovorans*.

**Figure 4:**
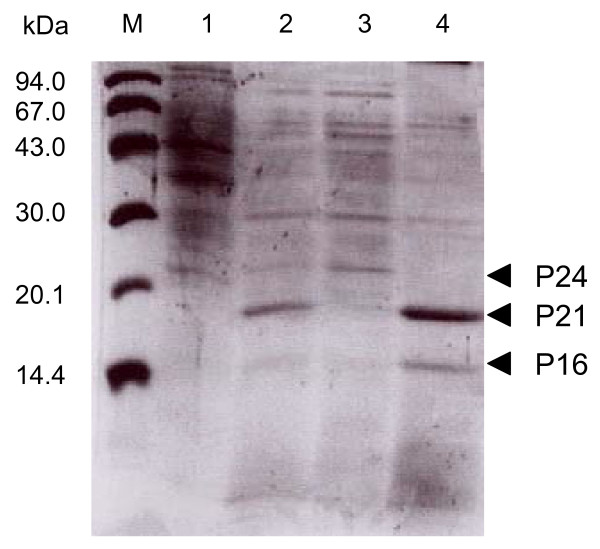
**Localization of P24, P21, and P16 in the cells**. Lanes are molecular weight markers, M; whole cell fraction cultivated in the absence of alkanes, 1; whole cell fraction cultivated in the presence of alkanes, 2. Soluble intracellular fraction after sonication of the cells, 3; insoluble membrane fraction after sonication, 4.

The effect of carbon chain length of alkanes on the induction levels of the proteins was examined. It is obvious that the induction effect increases in accordance with the increase in the chain length of alkanes (Fig. 3). It has previously been shown that strain B23 effectively degrades alkanes longer than dodecane [1]. These results strongly suggest that P24, P21, and P16 are related to the long-chain-alkane degradation by strain B23 or the production of these proteins was stimulated in the consequence of alkane degradation. Localization of the proteins in the cell was examined by fractionation of total cellular proteins (Fig. 4). Because P24 was recovered in a soluble fraction after disruption of the cells, this protein is probably a cytoplasmic protein. On the other hand, P21 and P16 were recovered in an insoluble form, suggesting that they are membrane proteins.

### Amino acid sequences of P21 and P16

The N-terminal amino acid sequences of P21 and P16 were determined as AFPLSGVGGFTISADLI (P21-N) and VPISGVGEFXVTFDKL (P16-N), respectively. These sequences, which are highly similar with each other, showed considerable similarity with that of cholesterol esterase from *Streptomyces lavendulae *[15]. Cholesterol esterase is a secretion enzyme which hydrolyzes long-chain fatty acid esters of cholesterol and mainly functions in mammalian tissues. In bacteria, only actinomycetes and pseudomonads [16] are reported to produce this enzyme.

### Cloning and analysis of genes encoding P21 and P16

Utilizing the information of N-terminal and internal amino acid sequences, 416 bp and 1.8 kb DNA fragments encoding a part of P21 and P16, respectively, were cloned and their nucleotide sequence was determined. The 416 bp fragment was confirmed to encode a part of P21 protein, because other internal amino acid sequences IDVVVTGGNVSGQNLK (P21-14) and TDNPLNVIDLSAPSLTLDK (P21-20) were encoded in the gene fragment. Finally, 603 bp and 588 bp open reading frames encoding 201 and 196 amino acid residues were obtained for P21 and P16, respectively. Both P21 and P16 were suggested to be secretion proteins that are anchored to the cell membrane, because possible signal peptides (38 and 45 amino acid residues, respectively) were found between the initiation methionine and the N-terminal amino acids of P21-N and P16-N. Although cholesterol esterase from *S. lavendulae *showed relatively high sequence similarity with the N-terminal sequence of P21 and P16, the similarity scores became negligible when it compared to the entire 201 and 151 amino acid residues (5.7 and 10< of E values, respectively in BLASTP, http://blast.ddbj.nig.ac.jp). This suggests that P21 and P16 would not be cholesterol esterase but unique membrane proteins probably responsible for the alkane uptake, tolerance, or degradation pathway in *G. thermoleovorans *cells. *Geobacilllus kaustophilus *HTA426 was isolated from deepest sea mud of the Mariana Trench and the genome sequence has been determined (NC_006510). When we look into its genome sequence, there are genes encoding orthologous proteins to P21 (YP_148623, 99% identical) and P16 (YP_148893, 94% identical). On the other hand, to our most surprise there is no corresponding gene in the genome of *G. thermodenitrificans*.

### Gene expression levels of P21 and P16

**Figure 5:**
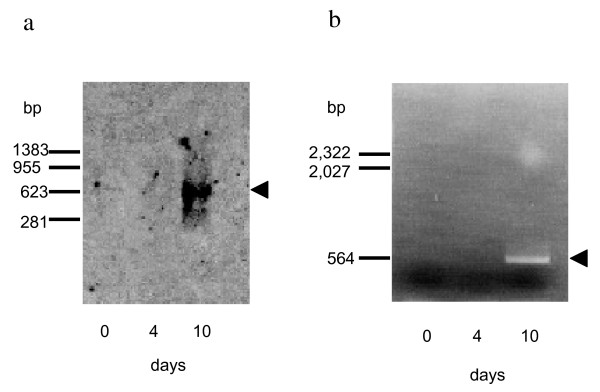
**Detection of mRNA for P21 and P16**. Total RNA sample was prepared from *G. thermoleovorans *B23 cultures before (indicated as 0 day in the figure) or after (4 and 10 days) induction by alkanes. Positive signals were indicated by arrowheads. a, Northern blot analysis of mRNA for P21. AlkPhos-labeled DNA probe gives signal at ca. 700 bases, where it hybridizes to mRNA for P21 on the membrane. b, Detection of mRNA for P16 by RT-PCR analysis.

Because P21 and P16, which are functionally unknown, were suggested to be membrane proteins, significant amount of these protein bands after 10 to 14-day cultivation on alkanes could be due to their accumulation in the dead cell membrane. In order to eliminate this possibility, production of P21 and P16 was examined at mRNA level. The results, which were obtained from Northern blotting experiment of P21, are shown in Fig. 5a. A strong signal was detected at an expected position of approximately 700 bases when RNA sample was prepared from the cells after 10-day culture. On the other hand, the probe DNA constructed for detecting mRNA of P16 hybridized with neither of RNA samples prepared, suggesting relatively short half-life of the mRNA. Then, RT-PCR method was adopted in this case, which is reported 10^6 ^times more sensitive than Northern hybridization method [17]. When the template RNA was prepared from 10-day culture, DNA fragment of expected size, ca. 500 bp, was clearly amplified by RT-PCR. The amplified DNA fragment was confirmed to be a part of P16 gene by determining the nucleotide sequence. No DNA amplification was observed for RNA samples prepared from 0 and 4-day cultures (Fig. 5b).

These results strongly suggest that the production of P21 and P16 was timely induced by alkanes at a transcription level. Because fatty acid, triacylglycerol, DCPK, and paraquat were no efficient inducer of P21 and P16 production, it is plausible that alkane molecules directly or indirectly control the transcriptional regulation of P21 and P16 genes.

### Amino acid sequence of P24

The N-terminal amino acid sequence of P24 was determined to be PFELPALPYPYDALEP (P24-N). This sequence was completely matched with that of superoxide dismutase (SOD) from strains in the genus *Geobacillus*. Cloning and sequencing of the entire gene encoding P24 revealed that it is a Mn-dependent type SOD of 204 amino acid residues, and showed 99.0% identical to Mn-SOD of *G. kaustophilus *HTA426 (YP_148310) or *G. stearothermophilus *(P00449) and 96% identical to *G. thermodenitrificans *NG80-2 (YP_001126490). The amino acid residues responsible for Mn binding, 76-GGXXXHXXE-84 and 49-QD-50, were completely conserved in P24.

### Detection of enzyme activities responsible for eliminating reactive oxygen molecules

**Figure 6:**
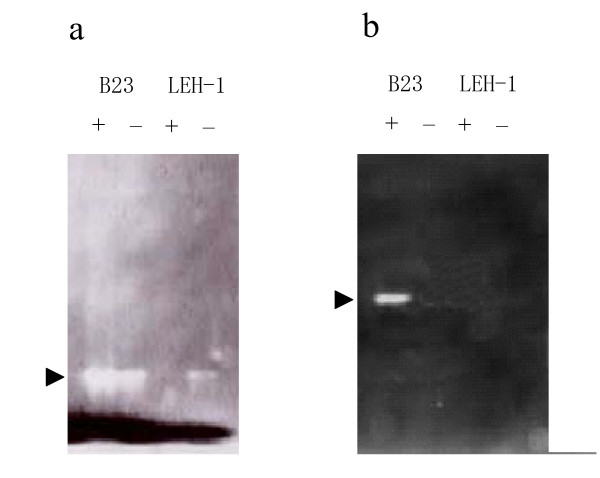
**Activity staining of SOD (a) and catalase (b)**. Crude cell extracts of *G. thermoleovorans *B23 and LEH-1 grown for 14 days on alkanes (+) and on 0.5% glucose (-) were separated on 7.5% native polyacrylamide gel. Arrows indicate respective enzyme activities.

SOD detoxifies superoxide anion to hydrogen peroxide, which in turn is generally broken down to water by the function of catalase or peroxidase. The B23 cells grown in the presence or absence of alkanes were tested for SOD, catalase, and peroxidase activity staining methods. The SOD activity of the B23 cells grown in the presence of alkane was slightly higher than that of the cells grown in the absence of alkanes as expected (Fig. 6a). It was found that catalase activity was detectable in the B23 cells only when they were grown on alkanes (Fig. 6b). When 0.5% glucose or glycerol was used as carbon source in the culture, the activities of SOD and catalase remained low. This observation indicates that these enzymes responsible for oxidative stress tolerance were produced as a result of not nutritional starvation (shift from nutrient L-broth to LBM mineral salts medium) but of alkane metabolisms. On the other hand, neither the SOD nor catalase was induced by alkanes in the *G. thermoleovorans *LEH-1 cells. Although it has been reported that LEH-1 showed relatively high peroxidase activity irrespective of the presence and absence of alkane in the media [18], this enzyme activity was not detectable level for both the B23 and H41 cells (figure not shown). Interestingly, SOD activity in LEH-1 cells with alkanes was disappeared in the presence of alkanes. This would have been occurred because SOD inducible oxygen molecules were mostly consumed by alkane degradation enzymes including acyl-CoA dehydrogenase and by regeneration of NAD^+^.

### Detection of acyl-CoA oxidase activity of B23

**Figure 7:**
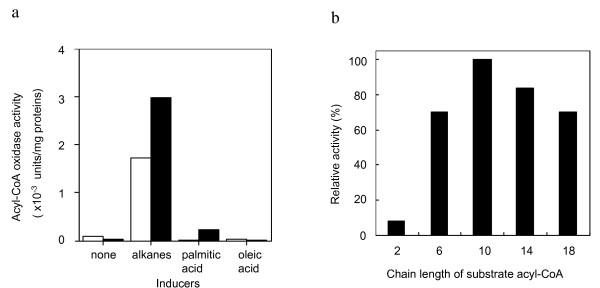
**Acyl-CoA oxidase activity of *G. thermoleovorans *B23**. a, Induction of acyl-CoA oxidase activity by alkanes or fatty acids. *G. thermoleovorans *B23 was cultivated in the presence of alkanes or single fatty acid at 70°C for 5 days (open bar) and 10 days (closed bar). Cells grown on simple LBM were used as a negative control. Acyl-CoA oxidase activity was measured using tetradecanoyl-CoA as a substrate. One unit was defined as the amount of enzyme producing 1 nmol of H_2_O_2 _in one min. b, Substrate specificity of acyl-CoA oxidase. Enzyme activity was compared each other using acyl-CoA with various alkyl chain length.

Both the SOD and catalase activities were specifically induced by alkanes at the time when strain B23 started to degrade alkanes. The SOD activity of *G. thermoleovorans *B23 cells was also inducible upon addition of paraquat in the medium, which generates superoxide anion (figure not shown). It seemed most likely that high SOD activity was required to detoxify superoxide anion, which was generated as a result of alkane degradation including oxidase reaction. So it is probable that a kind of oxidases catalyzes a step of alkane degradation pathway of *G. thermoleovorans *B23. Therefore, oxidase activity of the B23 cells was examined using tetradecane, tetradecanal, tetradecanol, or tetradecanoyl-CoA as a substrate. Increase in 500 nm (H_2_O_2 _formation) after the enzyme reaction was <0.01, 0.02, <0.01, and 0.16 for tetradecane, tetradecanal, tetradecanol, and tetradecanoyl-CoA, respectively. As far as we know, tetradecanoyl-CoA oxidase activity has never been reported for bacteria. As for acyl-CoA oxidase in bacteria, the gene encoding short chain acyl-CoA oxidase has been cloned from *Streptomyces fradiae*, which forms a biosynthetic gene cluster of macrolide antibiotic, tylosin [19]. In both the bacterial cells and mitochondria of eukaryotic cells, the first and rate-limiting step of β-oxidation pathway is catalyzed by acyl-CoA dehydrogenase, in which acyl-CoA is transformed to enoyl-CoA. This acyl-CoA dehydrogenase activity is replaced by acyl-CoA oxidase in eukaryotic peroxisome [20]. Peroxisome is an organella which generates and detoxifies reactive oxygen molecules like hydrogen peroxide or superoxide anions. According to the study of alkane degrading yeast *Candida*, peroxisome is highly developed in the cells grown on alkanes or fatty acids [21]. The development of peroxisomes in the cells of *C. tropicalis *grown on oleic acid was accompanied by high level expression of peroxisomal proteins, including acyl-CoA oxidase [13]. Catalase is also a marker enzyme of peroxisome and its activity in *Candida *cells grown on hydrocarbons was much higher than that in the cells grown on lauryl alcohol, glucose or ethanol. Although acyl-CoA oxidase is reported to increase in the *Candida *cells grown on fatty acids or organic acids, too, neither palmitic acid (hexadecanoic acid) nor oleic acid (octadecenoic acid) was an effective inducer for the production of acyl-CoA oxidase in *G. thermoleovorans *B23 (Fig. 7a). The acyl-CoA oxidase activity of strain B23 showed broad substrate specificity ranging from hexanoyl-CoA to octadecanoyl-CoA (Fig. 7b). Gene disruption experiments for P16, P21, P24 (SOD) and acyl-CoA oxidase have not been successful at this point to conclude that these enzymes are responsible for alkane degradation pathway of the strain. However, dramatic increase in the acyl-CoA oxidase, SOD, and catalase activities upon alkane degradation strongly suggests that acyl-CoA oxidase functions at the initial step of β-oxidation and resulting superoxide anion and hydrogen peroxide would be eliminated by cooperative reaction of SOD and catalase. This eukaryotic-type degradation mechanism of alkane in *G. thermoleovorans *cells might reflect chaotic living cell systems of common ancestor under high temperature condition of the primitive earth. Evolutional relationship between *G. thermoleovorans *and peroxisome in the eukaryotic cells are of great interest.

## Conclusion

We, for the first time, suggested that peroxisomal β-oxidation pathway exists in an extremely thermophilic alkane degrading *Geobacillus thermoleovorans *B23. This eukaryotic-type alkane degradation pathway in the bacterial cells might be a vestige of primitive living cell systems that would had evolved into eukaryotes.

## Methods

### Cells and plasmids

An extremely thermophilic alkane-degrading bacterium, *Geobacillus thermoleovorans *B23 was previously isolated from a deep petroleum reservoir in Minami-aga oil field (Niigata, Japan, [1]). *G. thermoleovorans *type strain LEH-1 (ATCC43513) was purchased from ATCC (American Type Culture Collection, Manassas, VA, [22]) and used as a comparative strain. *E. coli *DH5α was used as a host strain for the gene cloning with a cloning vector pCR2.1 (Invitrogen Corp., San Diego, CA).*E. coli *strain XL1-Blue MRA (P2) was used as a host strain for construction of a phage library of B23 genome.

### Culture media

Nutrient L-broth contained (per liter) 5 g of yeast extract (Difco, Detroit, MI), 10 g of Bacto-tryptone (Difco), and 5 g of NaCl (pH 7.2) was used for cultivation and storage of the strains. Cells were aerobically grown in a screw capped culture bottle without shaking at 70°C or 60°C for B23 and LEH-1, respectively. The bottle cap was opened once a day to avoid oxygen depletion. Solid medium was prepared by adding 1.5% agar or 4% gellan gum (Wako Pure Chemicals, Osaka, Japan). Mineral salts medium, LBM [23], was used for alkane degradation and protein induction experiments. LBM contained per liter; 0.25 g NaNO_3_, 0.25 g NH_4_Cl, 0.21 g Na_2_HPO_4_, 0.20 g MgSO_4_-7H_2_O, 0.09 g NaH_2_PO_4_, 0.04 g KCl, 0.02 g CaCl_2_, 1 mg FeSO_4_, 10 ml Trace mineral solution. Trace mineral solution contained per liter; 7 mg ZnSO_4_-7H_2_O, 1 mg H_3_BO_4_, 1 mg MoO_3_, 0.5 mg CuSO_4_-5H_2_O, 18 μg CoSO_4_-7H_2_O, 7 μg MnSO_4_-5H_2_O. Otherwise mentioned, LBM was supplemented with 1 ml (0.1%, v/v) single alkane or alkanes mixture (Standard gas oil, sulfur content 500 ppm level, Tokyo Chemical Industry, Tokyo, Japan). Alkanes used for cultivation substrate were filtrated (Millex-FG filter, pore size 0.2 μm, Millipore, Molsheim, France) for sterilization before use.

### Scanning electron microscopy

Cells before and after grown on alkanes were washed with 0.1 M K_2_HPO_4_/KH_2_PO_4 _buffer (pH 7.2) and fixed with 2.5% (v/v) glutaraldehyde in the same buffer for more than 2 h. Then, the cells were washed again with the buffer and dehydrated in acetone. After freeze-drying, specimens were coated with gold-palladium and observed with a Hitachi S-4700 scanning electron microscope (Hitachi, Tokyo, Japan).

### Induction of protein productions by alkanes

After aerobic cultivation in 1 L L-broth at 70°C for 24 h, B23 cells were harvested by centrifugation at 8,000 g for 10 min at 4°C and then washed with LBM. The cell pellet was suspended in 1 L LBM, which contained 0.1% (v/v) of alkane or standard gas oil. Bottles containing the suspension were closed tightly and incubated at 70°C for appropriate periods without shaking. Proteins induced by alkanes were analyzed by conventional SDS-12% polyacrylamide gel electrophoresis (SDS-PAGE, [24]). Soluble intracellular and insoluble membrane fractions of the cells were prepared by sonication (Branson sonifier model S-250, Branson Ultrasonics Corp., Danbrury, CT) and centrifuge (20,000 g for 30 min, 4°C).

### Amino acid sequence determination

The N-terminal amino acid sequences of the proteins were determined with a sequencing system Procise 491 (Applied Biosystems Japan, Tokyo, Japan). Samples were prepared by electro blotting the protein band from SDS-polyacrylamide gel onto a polyvinylidene difluoride (PVDF) membrane (Bio-Rad, Hercules, CA). Electroblotting was conducted at 50 mA for 1.5 h in a transfer buffer (10 mM CAPS, pH 11) containing 10% (v/v) methanol. Proteins were stained on the PVDF membrane with 0.1% Coomassie Brilliant Blue R in 40% (v/v) ethanol and 1% (v/v) acetic acid and destained for 1 min in 50% (v/v) ethanol. A strip of the membrane containing protein band was cut into pieces and subjected to the amino acid sequence analysis.

### Construction of B23 genomic DNA library

Total genomic DNA of *G. thermoleovorans *B23 was prepared as described previously [25] and was partially digested with *Sau*3AI. Then the DNA fragments were ligated with phage vector Lambda EMBL3 using Lambda EMBL3/*Bam*HI arm (Promega, Madison, WI) and packaged *in vitro *by Maxplax packaging extract kit (Epicentre Technologies, Madison, WI).

### Cloning of P24 (SOD) gene

Partial SOD gene was amplified by utilizing GeneAmp PCR System 2400 (Applied Biosystems Japan) with AmpliTaq DNA polymerase (Takara Bio, Shiga, Japan). PCR amplification primers used were designed based on N-terminal amino acid sequence determined in this work and a sequence of consensus region (Mn dependent type SOD of *B*. *stearothermophilus *193-VAKRYSEA-200, P00449). InstaGene Matrix (Bio-Rad) was used for preparation of genomic DNA.

In order to clone the entire SOD gene, inverse PCR method was adopted. Genomic DNA, which had been previously digested with *Sph*I (for subcloning of 5'-end) or *Acc*III (for subcloning of 3'-end), was self-ligated and used for template DNA. For the analysis of DNA fragments, agarose gel electrophoresis was performed under standard condition [23]. GeneClean kit (Bio 101, La Jolla, CA) was used to recover DNA fragment from agarose gel slices. The PCR amplified gene fragment was ligated independently into the cloning vector pCR2.1 (Invitrogen Corp.), with TA cloning kit (Invitrogen Corp.), and used for transformation of *E. coli *DH5α. Nucleotide sequence of the gene was determined by using ABI PRISM 310 genetic analyzer (Applied Biosystems Japan). The nucleotide and amino acid sequence of P24, Mn-SOD of strain B23, has been deposited in the EMBL/GeneBank/DDBJ under accession number BAA95631.

### Cloning of genes encoding P21 and P16

In order to clone the genes encoding P21 and P16, their internal amino acid sequences were determined as follows. Target proteins were prepared by slicing the SDS-PAGE gel and eluting out by vortex with 20 mM Tris-HCl (pH 8.0) containing 1% SDS overnight. After digestion of the protein with lysyl endopeptidase (LEP) under standard condition [26], each peptide fragment was fractionated by reverse phase HPLC (column: AQUAPORE RP300, 4.6 × 250 mm, Applied Biosystems Japan) and its N-terminal amino acid sequences was determined. Based on these amino acid sequences, PCR primers were constructed to amplify the target gene loci. A part of the gene encoding P21 was amplified by PCR with primers designed for N-terminal amino acid sequence, AFPLSGVGGFTISADLI (P21-N), and one of the internal amino acid sequences, PSLNTHYMSAGSITIPSMK (P21-37). B23 genome library was screened to obtain a phage clone containing the entire gene encoding P21. The nucleotide sequence of this gene and its flanking region has been submitted to EMBL/GenBank/DDBJ under accession number AB047106.

A part of the gene encoding P16 was amplified by, what we call, armed-PCR method using lambda EMBL3-B23 genomic DNA library as template DNA. The PCR amplification primers were designed for right arm of EMBL3 vector (5'-CGTCCGAGAATAACGAGTGGATC-3') and one of the internal amino acid sequences, AAQEFQTGADNITIDNGN (P16-16). The PCR amplified DNA fragments (1.8 kb) were ligated into the cloning vector pCR2.1. The complete nucleotide sequence was determined and found that the DNA fragment encodes a part of the P16 gene, including 5'-end. Utilizing this gene fragment as a probe, B23 genome library was screened to obtain a phage clone containing the entire gene encoding P16. The nucleotide sequence of this gene fragment has been submitted to EMBL/GenBank/DDBJ under accession number AB049820.

### Northern hybridization and RT-PCR

Cultures were taken from a bottle after 0, 4, and 10 days cultivation in the presence of alkanes. The cells were collected by centrifugation and frozen on dry ice. Total RNA was isolated at the same time by the method of Reddy *et al. *[27]. For Northern blot analysis, 20 μg each of total RNA was electrophoresed on 1% agarose gel containing formaldehyde as a denaturant. The RNA band was blotted onto a Hybond N^+ ^membrane (Amersham Pharmacia Biotech) using Transblot cell (Bio-Rad) under standard protocol. The PCR amplified 416 bp and 1.8 kb DNA fragments were used for detecting the mRNA of P21 or P16, respectively. Labeling the probe DNA, hybridization to the target mRNA, and detection of signals were performed using Gene Images AlkPhos direct labeling and detection system (Amersham Pharmacia Biotech) under standard protocols.

In order to analyze the transcription level of P16 gene, RT-PCR method was also adopted by using QIAGEN OneStep RT-PCR Kit (QIAGEN). Ten micrograms of total RNA sample was used as the initial template for RT-PCR in each case.

### Activity staining of superoxide dismutase (SOD)

Cell free extracts were prepared as follows; cells after cultivation in LBM supplemented with or without alkanes were washed and suspended with 50 mM K-phosphate buffer (pH 7.8), and then disrupted by sonication in ice bath. Cell disruption was monitored by microscopic observation at appropriate time interval. After a centrifugation at 15,000 g for 30 min (4°C), the resulting supernatant was subjected to gel electrophoresis using 7.5% non-denaturing polyacrylamide gel (pH 7.5)[24]. Then, the SOD activity was detected by negative staining method utilizing nitroblue tetrazolium [28].

### Activity staining of catalase

Cell free extracts were prepared and subjected to gel electrophoresis as mentioned above. Then, the gel was rinsed for 15 min three times with distilled water, soaked in a solution of 0.01 ml of 30% H_2_O_2 _in 100 ml water, and gently shaken for 10 min. The H_2_O_2 _solution was discarded and the gel was immediately rinsed with distilled water. A freshly prepared mixture of 30 ml each of 2% ferric chloride and 2% potassium ferricyanide was poured onto the gel for staining. The gel tray was gently but steadily rocked by hand over a light box. As soon as green color began to appear in the gel background, the ferricyanide mixture was rapidly removed and the gel was washed twice with water to terminate the coloring reaction [29].

### Measurement of oxidase activity

Oxidase activity was assayed by the method of Shimizu *et al*. [13]. The reaction mixture contained in 0.4 ml of 50 mM potassium phosphate buffer (pH 7.4), 0.33 μmol 4-aminoantipyrine, 4.24 μmol phenol, 0.004 μmol FAD^+^, 0.04 μmol substrate, 12 IU horseradish peroxidase (Sigma), and 0.1–0.2 mg cell free extract. Cell free extracts were prepared from the 14 days culture with 0.1% alkanes at 70°C. Although horseradish peroxidase is not stable under 70°C, we adopted this temperature for measuring thermophilic oxidase activity of strain B23. The reaction was carried out at 70°C for 10 min, and the production of H_2_O_2 _was measured by increase in absorbance at 500 nm. Tetradecane, tetradecanol, tetradecanal or tetradecanoyl-CoA was used as a substrate. Each value was an average of triple experiments and was subtracted that of negative control experiment without substrate.

## Abbreviations

bp: base pairs; kb: kilobase pairs.

## Authors' contributions

TK performed most of the experiments, analyzed the data and wrote the manuscript. AM helped TK with cultivation of B23 and preparation of protein samples. SK and MM were co-supervisors of TK and AM. All authors have read and approved the final version of the manuscript.
